# Single versus multiple reoperations for recurrent intracranial meningiomas

**DOI:** 10.1007/s11060-024-04673-8

**Published:** 2024-04-24

**Authors:** Francesco Maiuri, Sergio Corvino, Giuseppe Corazzelli, Marialaura Del Basso De Caro

**Affiliations:** 1 Department of Neurosciences and Reproductive and Odontostomatological Sciences, Neurosurgical Clinic, University “Federico II” of Naples, 80131 Naples, Italy; 2 Department of Advanced Biomedical Sciences, Section of Pathology, University “Federico II” of Naples, 80131 Naples, Italy

**Keywords:** Intracranial meningioma, Time to recurrence, Extent of resection, WHO grade, Ki67-MIB1, Multiple recurrences

## Abstract

**Purpose:**

To identify the risk factors and management of the multiple recurrences and reoperations for intracranial meningiomas.

**Methods:**

Data of a neurosurgical series of 35 patients reoperated on for recurrent intracranial meningiomas were reviewed. Analyzed factors include patient age and sex, tumor location, extent of resection, WHO grade, Ki67-MIB1 and PR expression at initial diagnosis, time to recurrence; pattern of regrowth, extent of resection, WHO grade and Ki67-MIB1 at first recurrence were also analyzed. All these factors were stratified into two groups based on single (Group A) and multiple reoperations (Group B).

**Results:**

Twenty-four patients (69%) belonged to group A and 11 (31%) to group B. The age < 65 years, male sex, incomplete resection at both initial surgery and first reoperation, and multicentric-diffuse pattern of regrowth at first recurrence are risk factors for multiple recurrences and reoperations. In group B, the WHO grade and Ki67-MIB1 increased in further recurrences in 54% and 64%, respectively. The time to recurrence was short in 7 cases (64%), whereas 4 patients (36%) further recurred after many years. Eight patients (73%) are still alive after 7 to 22 years and 2 to 4 reoperations.

**Conclusion:**

The extent of resection and the multicentric-diffuse pattern of regrowth at first recurrence are the main risk factors for multiple recurrences and reoperations. Repeated reoperations might be considered even in patients with extensive recurrent tumors before the anaplastic transformation occurs. In such cases, even partial tumor resections followed by radiation therapy may allow long survival in good clinical conditions.

## Introduction

Intracranial meningiomas often recur even after gross total resection with and without adjuvant radiation treatment, with a rate ranging from 10 to 32% at 10 years [1, 2]. The main risk factors include the WHO grade [3–5], the proliferation index Ki67-MIB1 [4, 6–8] and mitotic index [9], the extent of resection (EOR) according to Simpson [10, 11] and the postoperative adjuvant treatments [1, 12, 13]. Other investigated factors include patient age and sex [3, 4, 14, 15], tumor size [14, 16], location [17–21] and morphology [17, 22], brain invasion, progesterone receptor (PR) expression [4, 23–26].

While well-defined guidelines of treatment exist for intracranial meningiomas at first diagnosis [27], identifying the surgical resection as the gold standard of treatment for symptomatic meningiomas in good clinical conditions patients, the management of recurrences is more challenging, especially when occur many times, and often varies among Institutions. Most patients with recurrent meningiomas are cured after one reoperation and adjuvant radiation therapy; nevertheless, a lesser percentage experiences further recurrences even after many years. Although the extent of resection is mostly involved, the factors correlated to multiple recurrences have scarcely been investigated. The present study reviews the medical record data from a monoinstitutional surgical series of recurrent intracranial meningiomas with the aim to define the main risk factors for multiple recurrences and reoperations to assist the neurosurgeon and radiation therapist in the decision-making process of treatment and in the planning of neuroradiological and clinical follow-up.

## Methods

### Patient population

Data of a neurosurgical series of 560 patients operated on for intracranial meningiomas between June 2006 and December 2022 at University of Naples Federico II, have retrospectively been reviewed. Seventy-four (19%) were reoperations for recurrence in 48 patients. Inclusion criteria were patients who underwent reoperations, time to reoperation > 18 months, cases with complete surgical and pathological data of both primary and recurrent meningiomas. According to the inclusion criteria, 35 patients were eligible for the study. Sample was divided into two groups: group A (24 pts, 69%) with one recurrence and one reoperation; group B (11 pts, 31%), with two or more recurrences and reoperations.

### Analyzed factors and methods

The analyzed factors included patient age at initial observation and sex, meningioma location, Simpson grade [10] of resection at initial diagnosis, WHO grade, Ki67-MIB1 and progesterone receptor (PR) expression at initial diagnosis, pattern of regrowth, topography of recurrence, and extent of resection at first reoperation, WHO grade and Ki67-MIB1 between initial diagnosis and first recurrence, number of recurrences, management and outcome of patients with multiple recurrences and reoperations.

“Recurrence” was defined as the detection of a new meningioma at the dural site of the primary tumor after its gross total resection assessed intraoperatively or on the contrast-enhanced brain MRI performed 3 months after surgery.

Reoperation was offered to patients with symptomatic recurrence and in good clinical conditions.

Patients age at initial diagnosis was graded as < 65 years and ≥ 65 years. Meningioma location was classified as skull base, parasagittal-falx and brain convexity.

The WHO grade was defined by reviewing the histological specimens according to 2021 WHO Classifications [28].

Immunohistochemical studies were performed to evaluate the Ki67-MIB1 and PR expression.

The expression of Ki67-MIB1 was evaluated in all cases by using the monoclonal antibody MIB-1 Immunotech® (DAKO system) (dilution 1:1000, overnight incubation). The streptavidin-biotin system and the diaminobenzidine (DAB) were used for antigen detection and visualization. Ki67-LI count was performed by eye counting, taking the average on five adjacent representative fields of neoplastic cells in a hot spot area. The values of Ki67-LI were divided into three groups: group I 0–4%, group II 5–9%. Group III ≥10%.

The expression of PR was determined in all specimens with monoclonal antibody against the progesterone (DAKO 1:400, overnight incubation). The quantitative evaluation was expressed as percentage of positive nuclei among 100 cells, for a total of 500 cells. The percentage of PR positivity was graded as ≤ 30%, 31–60%, ≥61%.

Preoperative and at follow-up contrast-enhanced magnetic resonance imaging (MRI) studies of the brain were reviewed.

EOR was defined according to Simpson grade on the surgeon’s assessment during surgery and confirmed by a post-contrast MRI of the brain one to three months after surgery.

The pattern of regrowth and topography of the recurrences at first reoperation were classified according to our previous proposal system [29, 30], as: (1) Localized, inside of the area of the previously resected tumor; (2) Peripheral, inside and outside within 1 cm from the original tumor margins; (3) Multicentric, with multiple nodules both at previous attachment and distal, with seemly health interposed dura; (4) Diffuse, with diffuse dural infiltration both localized and distant.

WHO grade and Ki67-MIB1 of the meningioma at the first recurrence, as compared to the initial surgery, were defined as “stable” (in the same WHO grade and KI67-MIB1 subgroup) or “progression” (WHO grade I to II, from lower to higher Ki67 values).

All the above cited analyzed factors were statistically compared between group A and B.

### Statistical analysis

The study utilized contingency tables, Fisher’s exact test, Linear regression analysis and One-Way ANOVA to calculate descriptive statistics. Univariate analysis was performed to find predictors of outcome for each independent variable: linear regression analysis for continue variables, and simple logistic regression for binary variables. Only variables with a p-value < 0.2 from the univariate analysis were included in the multivariate regression model, to avoid overfitting. Multivariate analysis was performed using the Cox proportional hazards model to assess the contribution of predictor variables. The threshold for statistical significance was set at a p-value of 0.05. The data were aggregated in Microsoft Excel (version 14.2.5), and statistical analysis was performed using GraphPad software (version 10.0).

## Results

### Demographic, radiological and surgical data (Table 1)

The 35 enrolled patients included 22 females (63%) and 13 males (37%). According to the age at initial surgery, 20 (57%) were younger than 65 years and 15 (43%) were 65 years old or older. The age ranged from 34 to 73 years (median 59 years). Patients with multiple reoperations, as compared to those with single reoperation, were mainly males (*p* = 0.0005) and aged < 65 years old (*p* = 0.0013).

Meningioma location was on the skull base in 10 patients (28.5%), in the parasagittal-falx region in 17 (48.5%), on the brain convexity in 7 (20%) and in the lateral ventricle in one (3%). The statistical analysis of meningioma location revealed significant tendency for parasagittal-falx meningiomas to experience a single reoperation (group A) (*p* = 0.01); on the other hand, no difference of number of reoperations was observed among skull base and brain convexity meningiomas.

The EOR at initial surgery was of Simpson grade I in 7 patients (20%), grade II in 10 (28.5%), grade III in 14 (40%), and grade IV in 4 (11.5%). Grades I and II resections were more frequently observed in the single reoperations group (group A) (64% vs. 46%). The difference was statistically significant (*p* = 0.0045).

Radiation therapy after initial surgery was administered in 15 patients and included cases with WHO grade I with subtotal resection and WHO grade II meningiomas.

### Pathological findings (Table 2)

The eleven patients with WHO grade 2 meningiomas had no significantly different number of reoperations than the 24 patients with WHO grade 2 tumors (*p* = 0.44).

The Ki67-MIB1 was ≤4% in 11 cases (31%), between 5% and 9% in 9 (26%) and ≥10% in 15 (43%).

No statistically significant difference between group A and B was observed according to Ki67-MIB1 values (*p* = 0.65). To better investigate the higher rates of multiple reoperations in younger male patients, we have studied the Ki67-MIB1 in males. The correlation with patient sex has show significantly lower rate of values ≤ 4% (15%) and higher rate of values ≥10% (62%), as compared to the females (41% and 32%, respectively) (*p* < 0.0001). On the other hand, no significant differences of Ki67-MIB1 values were observed between patients aged < 65 years and ≥65 years.

The PR expression was ≤30% in 25 patients (70%), between 31 and 60% in 5 (15%) and > 61% in 5 (15%). No statistically significant difference was observed between group A and B according to PR expression values (*p* = 0.31).

### Recurrence related findings (Table 3)

The 24 patients (69%) who experienced one recurrence and one reoperation did not show significantly different median time to recurrence than the 11 (31%) who underwent two or more reoperations (*p* = 0.34). According to the topographic pattern of recurrence, the 23 patients who had localized-peripheral recurrences at the first reoperation had significantly lower rates of multiple reoperations (27%) than 12 patients with multicentric-diffuse pattern of recurrences (73%). This finding is strongly significant (*p* < 0.00001).

The extent of resection at first reoperation is also important. A gross-total resection (GTR) was achieved in 26 patients (73%) and a subtotal (STR) in 9 (27%). Among the 24 patients who experienced a single reoperation, 22 (91%) underwent GTR versus only 4 among the 11 patients (36%) belonging to the group of multiple recurrences (*p* < 0.00001). The radiotherapy was administered after first reoperation in the 20 patients who had not been treated before.

The WHO grade of meningiomas at first reoperation was similar to the initial surgery in 31 patients (89%) and showed progression from 1 to 2 in 4 (17%), with no difference between single and multiple reoperations groups (*p* = 0.64).

The Ki67-MIB1 at first reoperation was similar to the initial surgery (in the same subgroup) in 25 patients (70%) and showed progression in 10 (30%), with no significant correlation with the number of reoperations (*p* = 0.87).

### Management and outcome of patients with multiple recurrences (Table 4)

Among the 11 patients of group B, 4 (36%) had 2 reoperations, 5 (46%) had 3 reoperations and 2 (18%) had 4 reoperations. The time to further reoperations, as compared to time to first recurrence, was reduced in 7 patients (64%) and almost similar or increased in 4 (36%).

The WHO grade at further reoperations increased in 6 cases (54%) (from 1 to 2 in three and from 2 to 3 in in three) and was similar to the initial grade in 5 (46%) (all WHO grade 2).

The Ki67-MIB1 was stable (in the same subgroup) in 4 cases (36%) and increased in 7 (64%).

No postoperative death occurred. One patient (9%) experienced infection of the craniotomy site.

The follow-up ranges from 7 to 22.3 years (median 16.8 years). Eight patients (72%) are alive with stable residual tumor and no adjunctive clinical deficits in 7; one with invasive spheno-orbital meningioma shows right proptosis and amaurosis and tumor progression. Three patients died for tumor progression after a survival of 7 to 15 years.

### Statistical analysis

The simple logistic regressions showed the Simpson grade was statistically involved in early meningioma recurrence (Z = 2.269; *p* = 0.02).

Multivariate analysis using Cox regression showed that none of the variables were independently associated to the multiple recurrence rate (Table 5).

## Discussion

Intracranial meningiomas sometimes present multiple recurrences even in a long time after the initial surgery. Thus, patients operated on for a recurrent meningioma ask to the neurosurgeon whether they are definitively cured after the first reoperation and adjuvant radiation therapy or are at risk of further recurrences and reoperations. The present study attempt to answer to this question through a detailed retrospective analysis of many demographics, histopathological, neuroradiological and surgical risk factors. No other study has discussed these features correlated to the number of reoperations of intracranial meningiomas.

The unexpected higher frequency of multiple reoperations in younger male patients deserves to be discussed. Younger patients are at higher risk to develop multiple recurrences over the years than older ones, because the longer life expectancy. On the other hand, elderly patients have a shorter follow-up, due to the higher risk of death for other causes. The higher incidence of multiple reoperations among males agrees with the more aggressive behavior of meningiomas in male sex. The correlation between sex and Ki67-MIB1 at initial diagnosis has shown in male patients significantly lower rate of cases with values ≤4% (15%) and higher rate (62%) of values ≥10% as compared to the females (41% and 32%, respectively) (*p* < 0.00001). On the other hand, the Ki67 values of the overall series are not correlated with the number of recurrences.

The meningioma location is a relevant factor for recurrence [19, 31, 32]. As shown in our previous report [19], the recurrence rates of non-skull base [17, 33] and lateral skull base meningiomas [34, 35] are significantly higher than that of medial skull base ones [36, 37]. This may reflect the different embryological origin of the meninges and biomolecular expression of the meningiomas according to their location [38–40]. In this study a correlation between initial location and number of recurrences was evidenced only for parasagittal-falx meningiomas, whereas the data of other locations are not significant. This suggests that the number of recurrences mainly depends on the topography of the recurrent tumor more than the initial location.

The extent of resection at initial surgery according to Simpson grade is a well-recognized factor correlated to the recurrence of meningiomas [1, 10, 11, 37, 41]. This study shows that this is a risk factor also for multiple recurrences. Grades III or IV resections were more significantly correlated to multiple recurrences and reoperations than grades I and II ones (*p* = 0.045). Residual intrasinusal, bone and intradural tumor is more likely to progress and escape to the resection at the first reoperation. Although the term “gross-total resection” is often used to define Simpson grades I-II-III together, most cases of grade III resections, as in our study, have different prognostic values than grades I and II.

The lack of correlation between pathological findings at initial diagnosis and multiple reoperations is an interesting result of our study. The proliferation index of the meningioma at initial diagnosis, defined as mitotic index using anti-pHH3 antibody [9] or as Ki67-MIB1 [4, 7, 24], was significantly correlated with the recurrence and also with the recurrence-free survival [8]. Most studies use a Ki67 cut-off at 4% to differentiate between high and low risk of recurrence. We did not find correlation with the number of reoperations also between Ki67 values (5–9% versus ≥10%) of atypical meningiomas. Three other studies focusing on multiple reoperations [42–44] do not include data of Ki67-MIB1 of both initial diagnosis and recurrences.

Many studies have focused on PR expression and recurrences of meningiomas; some [23–25, 45] and two by our group [4, 26] have found significant inverse correlation, with higher recurrence rates in meningiomas with low PR expression at initial surgery. The present study first correlates the initial PR expression with the number of reoperations; the results do not evidence significant differences between patients who experienced single and multiple reoperations.

Meningiomas may recur with different patterns of growth, as first described in our previous reports [29, 30]. Most recur at the previous dural site (“localized”) or inside and outside the original tumor margins within 1 cm (“peripheral”); some may recur in multicentric and diffuse forms, with multiple nodules, even distant, and interposed seemly normal (“multicentric”) or infiltrated (“diffuse”) dura. Multicentric and diffuse recurrences represent the progressive growth of multiple distant dural tumoral nodules with different potential of growth [46, 47]. In our previous studies [29, 30], patients with multicentric-diffuse recurrences, when compared with those with localized-peripheral recurrences, showed significantly higher rates of flat-shaped tumors and ki67-MIB1 > 4% and lower rates of gross-total resections. In the present study, the multicentric and diffuse patterns of regrowth at the first recurrence are significantly associated to multiple reoperations (*p* < 0.00001). Two recent studies [48, 49] focus on the pattern of recurrence of meningiomas with the aim to guide the surgical resection and adjuvant therapy, but they do not correlate them with further recurrences and patient outcome.

The rate of gross-total resections at the first reoperation is also significantly lower in patients who later required multiple reoperations. This is a consequence of the significantly higher rate of multicentric-diffuse patterns of regrowth and recurrences. This confirms that the gross-total resection at the first reoperation is the most important factor for obtaining patient cure and no further regrowth.

In this study the histological findings (WHO grade and Ki67-MIB1) of further recurrences, as compared to those at the first reoperation, show progression in about half of the cases; however, only 3 underwent anaplastic transformation (WHO grade 3), whereas others were atypical (WHO grade 2) also after two or more reoperations. This suggests the need for reoperating on the further recurrences early, before the tumor becomes anaplastic.

Lemeé et al. [44] found in their series that the time to retreatment decreased significantly between surgeries in patients requiring repeated resections. In our study, this occurred in 7 among 11 patients (64%) who had multiple reoperations; on the other hand, in 4 others the second reoperation occurred later than the first one. This may probably result from late histological progression.

The neurosurgeon must often decide when reoperate on meningiomas which again recur after the first reoperation and radiation treatment. Although the WHO grade and proliferation index often increase in further recurrences, in many patients they remain almost unchanged. Surgery should be considered in symptomatic patients, even with extensive recurrent tumor, mainly not elderly and without significant comorbidities. Asymptomatic young patients with further recurrences after the first reoperation should also be reoperated on, mainly if harboring large recurrences in less critical regions. In such cases even partial tumor resections in repeated operations, followed by radiosurgery may allow long survival in good clinical conditions.

### Limitation of the study

The retrospective nature of the study and the small size of the sample of patients included, represent the main limitations of the study.

## Conclusion

The pattern of regrowth and the extent of resection at first recurrence are the most important risk factors for multiple reoperations of intracranial meningiomas. The extent of resection at initial surgery is also significant. On the other hand, the pathological findings at initial diagnosis, although well-recognized risk factors of recurrence, are not correlated to the number of reoperations. Biomolecular studies will better define this aspect. The often-late anaplastic transformation must suggest early re-reoperation for further recurrences.

**Table 1:** Demographic, radiological and surgical data and number of recurrences

Covariation	Overall series 35 pts	Single recurrence 24 pts (69%)	Multiple recurrences 11 pts (31%)	Statistical significance (p value)
Age				
• < 65y	20 (57%)	12 (50%)	8 (73%)	*p* = 0.0013
• ≥ 65y	15 (43%)	12 (50%)	3 (27%)	
**Sex**				
• F	22 (63%)	17 (71%)	5 (46%)	*p* = 0.0005
• M	13 (37%)	7 (29%)	6 (54%)	
**Meningioma location**				
• skull base (SB)	10 (28.5%)	6 (25%)	4 (36%)	*p* = 0.12
• parasagittal-falx (PF)	17 (48.5%)	13 (54%)	4 (36%)	*p* = 0.01
• brain convexity (BC)	7 (20%)	4 (17%)	3 (28%)	*p* = 0.08
• lateral ventricle (LV)	1 (3%)	1 (4%)	---	
				SB vs. PF vs. BC:
				*p* = 0.016
**Extent of resection at initial surgery (Simpson grade)**				
• I	7 (20%)	5 (24%)	2 (18%)	I-II vs. III *p* = 0.03
• II	10 (25.5%)	8 (33%)	2 (18%)	I-II vs. IV *p* = 0.007
• III	14 (40%)	9 (38%)	5 (46%)	I-II vs. III-IV *p* = 0.0045
• IV	4 (11.5%)	2 (8%)	2 (18%)	

**Table 2:** Pathologic findings at initial diagnosis and number of reoperations

Covariation	Overall series (35 pts)	Single recurrence 24 pts (69%)	Multiple recurrences 11 pts (31%)	Statistical significance (p value)
WHO Grade				
• I	11 (31%)	8 (33%)	3 (27%)	*p* = 0.44
• II	24 (69%)	16 (67%)	8 (73%)	
**Ki67 MIB1**				
• I: 0–4%	11 (31%)	8 (33%)	3 (27%)	*p* = 0.44
• II: 5–9%	9 (26%)	6 (25%)	3 (27%)	*p* = 0.87
• III: ≥ 10%	15 (43%)	10 (42%)	5 (46%)	*p* = 0.66
				I vs. II vs. III: *p* = 0.65
**PR expression**				
• I: ≤ 30%	25 (70%)	18 (75%)	7 (64%)	*p* = 0.12
• II: 31–60%	5 (15%)	3 (12.5%)	2 (18%)	*p* = 0.43
• III: > 61%	5 (15%)	3 (12.5%)	2 (18%)	*p* = 0.43
				I vs. II vs. III: *p* = 0.31

**Table 3:** Recurrence-related findings and number of reoperations

Covariation	Overall series (35 pts)	Single recurrence 24 pts (69%)	Multiple Recurrences 11 pts (31%)	Statistical significance (p value)
**Time to recurrence (median)**	47 mo	48 mo	57 mo	*p* = 0.34
**Topography of the recurrence at the first reoperation**				
• localized-peripheral	23 (66%)	20 (83%)	3 (27%)	*p* < 0.00001
• multicentric/diffuse	12 (34%)	4 (17%)	8 (73%)	
**Extent of resection at first reoperation**				
• gross-total	26 (73%)	22 (91%)	4 (36%)	*p* < 0.00001
• subtotal	9 (27%)	2 (9%)	7 (64%)	
**WHO grade and Ki67 MIB1 between initial surgery and first reoperation**				
WHO grade				
• stable	31 (89%)	21 (87.5%)	10 (91%)	
• progression	4 (17%)	3 (12.5%)	1 (9%)	
Ki67 MIB1				
• stable	25 (70%)	17 (71%)	8 (73%)	*p* = 0.87
• progression	10 (30%)	7 (29%)	3 (27%)	

**Table 4:** Management and outcome of the 11 patients with multiple recurrences

Covariation	N. of patients (%)
**Number of reoperations**	
• 2	4 (36%)
• 3	5 (46%)
• 4	2 (18%)
**Time to further reoperations**	
• decreased	7 (64%)
• similar/increased	4 (36%)
**Postoperative complications**	
• death	---
• infection of craniotomy site	1 (9%)
**WHO grade at further recurrences**	
• stable	5 (46%)
• progression	6 (54%)
**Ki67-Li at further recurrences**	
• stable	4 (36%)
• progression	7 (64%)
**Follow-up (years)**	7-22.3 years Median (12 years)
**Outcome**	
• alive-stable	7 (64%)
• alive with progression	1 (9%)
• dead	3 (27%) (7–15 years)

**Table 5:** Simple logistic regression analysis between single (= 0) and multiple (= 1) reoperation and categorical independent variables

Model	Missing data		Std. Error		Censored (0)		Z		p-value		AUC	p-value
Patient age	0		0.096		20		1.24		0.21		0.6	0.31
Sex	0		0.10		22		1.419		**0.15**		0.61	0.25
Location*	0		0.098		14		1.877		**0.06**		0.65	0.09
WHO grade	0		0.105		11		0.35		0.72		0.53	0.77
Simpson grade	0		0.091		17		2.269		**0.02**		0.69	**0.05**
PR expression	0		0.110		25		0.687		0.49		0.56	0.58
Ki67-MIB1	0		0.10		20		0.210		0.83		0.51	0.86
Multivariate Cox proportional hazards regression analysis– only variables with *p* < 0.2 from univariate analysis were included												
Variable		**Estimate HR**		**95% CI**		**Std. Error**		**Z**		**p-value**		
Age		3,115		0,5342 to 20,63		0,908		2.723		0.27		
Location*		1,000		0,09634 to 10,38		1,134		1,974		0.45		
Simpson		0,1333		0,009325 to 1,591		1,252		3,447		0.15		

* Location is expressed as continue variable (SB = 1; PF = 2; BC = 3; LV = 4) # Sex is expressed as binary variable (1 = M; 0 = F) ¥WHO grade is expressed as binary variable (1 = WHO grade I; 2 = WHO grade 2)

## Data Availability

Data of the current original research are available from the corresponding author on reasonable request.
